# Breast cancers as ecosystems: a metabolic perspective

**DOI:** 10.1007/s00018-023-04902-9

**Published:** 2023-08-10

**Authors:** Flavia Martino, Mariadomenica Lupi, Enrico Giraudo, Letizia Lanzetti

**Affiliations:** 1 Department of Oncology, University of Torino Medical School, Turin, Italy; 2 Candiolo Cancer Institute, FPO-IRCCS, Candiolo, Turin, Italy; 3 Department of Science and Drug Technology, University of Torino, Turin, Italy

**Keywords:** Metabolism, Glycolysis, Microenvironment, Metastasis, Adipocytes, Microbiota

## Abstract

Breast cancer (BC) is the most frequently diagnosed cancer and one of the major causes of cancer death. Despite enormous progress in its management, both from the therapeutic and early diagnosis viewpoints, still around 700,000 patients succumb to the disease each year, worldwide. Late recurrency is the major problem in BC, with many patients developing distant metastases several years after the successful eradication of the primary tumor. This is linked to the phenomenon of metastatic dormancy, a still mysterious trait of the natural history of BC, and of several other types of cancer, by which metastatic cells remain dormant for long periods of time before becoming reactivated to initiate the clinical metastatic disease. In recent years, it has become clear that cancers are best understood if studied as ecosystems in which the impact of non-cancer-cell-autonomous events—dependent on complex interaction between the cancer and its environment, both local and systemic—plays a paramount role, probably as significant as the cell-autonomous alterations occurring in the cancer cell. In adopting this perspective, a metabolic vision of the cancer ecosystem is bound to improve our understanding of the natural history of cancer, across space and time. In BC, many metabolic pathways are coopted into the cancer ecosystem, to serve the anabolic and energy demands of the cancer. Their study is shedding new light on the most critical aspect of BC management, of metastatic dissemination, and that of the related phenomenon of dormancy and fostering the application of the knowledge to the development of metabolic therapies.

## Introduction

The emerging picture of cancers as ecosystems, and the role of metabolism in their establishment, is spurring growing interest in the metabolic features of cancer and how they can be exploited to identify vulnerabilities to enhance our therapeutic abilities and benefit cancer patients. As multiomics orthogonal approaches unravel the complexity and the heterogeneity of cancer ecosystems, metabolic plasticity is being increasingly recognized as a hallmark of cancer that intersects cancer-intrinsic and -extrinsic features that concur to determine its evolvability [1, 2]. These general features are, however, differently instantiated in distinct types of tumors, where local, organ-specific conditions determine the evolutionary trajectory of individual cancers. Thus, a higher level of resolution of metabolic alterations, focused on individual types of cancer, is needed.

Breast cancer (BC) is the most frequently diagnosed cancer worldwide, accounting for ~ 12% of all cancer diagnosis and ~ 7% of cancer related deaths yearly [3]. BCs are phenotypically and molecularly heterogeneous [4, 5]. This complexity is captured by a widely used clinical classification relying on the expression of hormone receptors (estrogen receptor, ER, and progesterone receptor PGR) and of the oncogenic receptor tyrosine kinase ERBB2 (also known as HER2). Tumors characterized by positivity for hormone receptors are called Luminal, further subclassified into Luminal A or B subtypes, based on their proliferative index [4]. HER2 BCs, display amplification and overexpression of ERBB2. Finally, triple-negative BCs (TNBC) are negative for ER, PGR and HER2 [4]. Extensive molecular profiling, based on transcriptomic analysis, has confirmed the distinct molecular features of these subtypes and added valuable prognostic sub-stratification, especially in ER + tumors, which represent ~ 65% of all BCs. These molecular classifications and the derived prognostic algorithms have relevant clinical impacts on therapeutic decision-making [6].

BCs represent a particularly valuable setting to investigate the impact of metabolic alterations.

First, despite their heterogeneity they share several starting microenvironmental conditions, such as organ architecture and composition of resident normal cellular populations. This might, in principle, aid the understanding of how cell-autonomous (genetic, epigenetic, or functional) and non-cell-autonomous alterations influence the metabolic phenotypes of BC cells and normal mammary cells.

Second, because of the diffusion of population screening for early diagnosis, many BCs are detected at a relatively initial stage of their natural history. This means that early lesions (essentially ductal carcinoma in situ*,* DCIS) are available for studies aimed at dissecting molecular determinants of cancer progression, including metabolic alterations.

Third, the ER + BCs which are not cured by surgery and adjuvant therapy can recur as clinical metastases frequently after long periods of remission (10–20 years). Thus, BC is a good model for studying the phenomenon of metastatic dormancy, which is not only a mysterious aspect of the metastatic phenotype but also one of the major causes of concern in the clinical management of cancer patients.

Fourth, the mammary gland is one of the most dynamic organs in the animal kingdom that repeatedly undergoes rounds of expansion and involution. This feature is thought to promote the high oncogenic potential of the breast by increasing the risk of incurring genetic alterations. Moreover, mammary epithelial cells are primed to expand exploiting a metabolic plasticity similar to cancer cells [7].

Finally, BCs display some unique features in the cancer–microenvironment interaction. The first feature is related to the cancer–adipocyte interaction. The breast is composed essentially of adipose and fibro-glandular tissues at an approximate ratio of 9:1. Breast adipocytes play a critical role in the development, maintenance, and remodeling of the mammary gland [8, 9]. Similarly, adipocytes play a critical role in the BC microenvironment, including induction of a pro-tumorigenic inflammatory state, stimulation of proliferative and pro-metastatic phenotypes, and metabolic reprogramming [10, 11]. The second feature concerns the interaction between BC cells and the microbiota. Also in this case, some unique features of the BC microenvironment and of its microbiota contribute to the evolvability of these cancers.

In this review, we will summarize recent advances in the understanding of BCs as metabolic ecosystems with the intent of illustrating how this knowledge can have an impact on the management of BC patients.

## General aspects of metabolic plasticity in cancer

In this section, we provide some general information on the major circuitries and mechanisms that characterize cancer metabolism, from the viewpoints of the cancer cell (cell-autonomous effects) and of the tumor microenvironment (TME) (non-cell-autonomous effects). A few exceptions aside, this distinction is somewhat arbitrary, in line with the idea that in a co-evolving ecosystem the two components could not exist per se.

### The cancer cell viewpoint

Cancers have high metabolic demands aimed at sustaining anabolic processes to build up biomass for cellular duplication and catabolic processes to meet energetic needs. These needs are faced with the remarkable challenge posed by the high proliferation rate of tumors that leads to a severe mismatch between the tumor mass and its vascularization, with ensuing hypoxia and scarcity of nutrients. Hypoxia in itself is a major driver of metabolic adaptation, as it induces the activation of the HIF-1 (Hypoxia-Inducible Factor-1) transcriptional complex [12] that regulates the expression of genes involved in neo-angiogenesis of the tumor, metabolic reprogramming, redox homeostasis, epithelial-to-mesenchymal transition (EMT) and cancer stem cell specification, and remodeling of the extracellular matrix (ECM). We will discuss some of these phenotypes in the remainder of this review, while referring to other reviews for in-depth accounts [13–15]. Of note, several genetic alterations in cancer that impinge on cancer metabolism also activate HIF-1 or converge on the actuation of the same metabolic pathways, thereby enforcing feed-forward loops toward cancer metabolic adaptation [16].

**Fig. 1:**
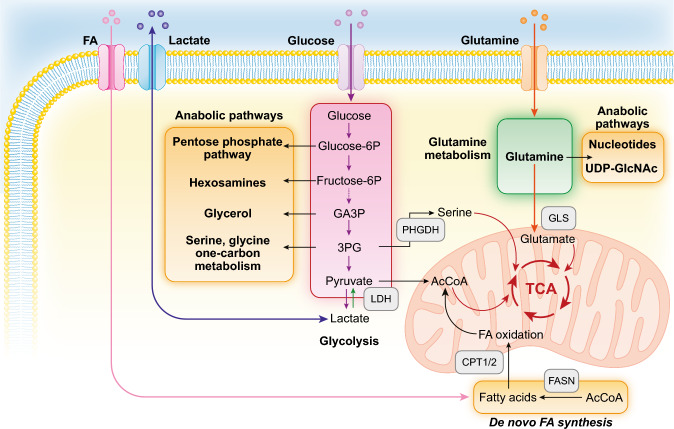
Major metabolic pathways altered in cancer. The figure illustrates some of the metabolic pathways discussed in the main body, and it is not comprehensive of all reactions (for instance, in the glycolytic pathway only key reactions leading to anabolic pathways are shown). Some of the key enzymes are shown in the gray boxes. *FA* fatty acid *GA3P* glyceraldehyde 3-phosphate, *3PG* 3-phosphoglycerate, *LDH* lactate dehydrogenase, *PHGDH* phosphoglycerate dehydrogenase, *AcCoA* acetyl coenzyme A, *CPT1*/2 carnitine palmitoyltransferase 1 and 2, *TCA* tricarboxylic acid cycle, *GLS* glutaminase, *FASN* fatty acid synthase, *UDP-GlcNAc* uridine diphosphate *N*-acetylglucosamine

The best characterized metabolic alteration in cancer cells is the preference to reduce pyruvate to lactate: something that cancer cells do even in the presence of oxygen. For this reason, the process is called aerobic glycolysis and it is also known as the Warburg effect [17, 18] (Fig. 1). The phenomenon was interpreted, for a long time, as an expedient enacted by cancer cells to circumvent a putative defect in mitochondrial oxidative phosphorylation (OXPHOS), by obtaining ATP from the pyruvate to lactate conversion. Today, it is well established that mitochondria in cancer cells are not only functional but also indispensable for tumor survival, both as producers of energy and of anabolic intermediates in the tricarboxylic acid cycle (TCA) [18–21]. The Warburg effect is, therefore, being re-interpreted as a means to increase glycolytic flux to heighten the availability of intermediates to feed anabolic pathways such as the pentose phosphate pathway, the hexosamine pathway, glycerol biosynthesis, and serine–glycine–one-carbon metabolism (Fig. 1). The metabolic advantage provided by the Warburg effect is not exclusively cell-autonomous, as it can trigger, in the TME, cancer-favorable circuitries involving normal stromal cells. This is the case, for instance, of the so-called reverse Warburg effect in which cancer cells stimulate aerobic glycolysis in the surrounding stroma. In turn, glycolytic stromal cells secrete high-energy metabolites, such as pyruvate or lactate, thereby sustaining the TCA cycle and OXPHOS metabolism in cancer cells [22].

A consequence of the Warburg effect is the high production of lactate that is released in the TME. Long regarded simply as a waste product, lactate is now considered a critical “oncometabolite.” The pleiotropic actions of lactate (both in the cancer cell and in the cellular TME) are exerted in four major ways: (i) as a high-energy carbon donor for anabolic synthesis, (ii) as an inducer of microenvironmental acidification, (iii) as a signaling molecule through its ability to activate the G-protein coupled receptor GPR81 [23–25], and iv) as a post-translational modification and epigenetic modifier, when appended to protein substrates, such as histones, in the process of lactylation [26]. While we refer the reader to comprehensive reviews on the topic [27–31], we will touch again on some aspects of the lactate relevance in the TME in the following paragraphs.

Amino acid metabolism is also critical to sustain the high metabolic demands of cancer cells. Glutamine metabolism is certainly the most relevant one, as it supports several anabolic and energetic needs (Fig. 1): (i) replenishing the TCA cycle through an anaplerotic reaction leading to the synthesis of α-ketoglutarate (α-KG), (ii) supporting the synthesis of nucleotides and non-essential amino acids (NEAAs), (iii) supporting the synthesis of glutathione which, in turn, is needed for the maintenance of the redox state through neutralization of reactive oxygen species (ROS), and (iv) favoring the capture of essential amino acids through an antiport-mediated exchange mechanism [32–34]. As a consequence, different types of cancer cells, including BC cells, are addicted to glutamine [35, 36].

A sufficient supply of other amino acids is also critical for the metabolic economy of cancer cells. Serine, for instance, is involved in anaplerotic replenishment of the TCA and in the one-carbon pathway [37]. Cancer cells ensure a sufficient supply of amino acids through various mechanisms: (i) expression of specific membrane transporters [38], (ii) scavenging of extracellular proteins by macropinocytosis [39], (iii) digestion of the ECM [40, 41], and (iv) activation of autophagy in the cancer cell or in stromal fibroblasts which then release the catabolized amino acids in the interstitium for utilization by cancer cells [42–44]. In the following sections, we will return to different aspects of amino acid metabolism in relation to BC.

Finally, cancer cells heavily rely on lipids, in particular on fatty acids (FAs), for anabolic and catabolic needs. FAs serve multiple purposes as they (i) are needed for the formation of biological membranes, (ii) provide energy storage and can be mobilized for fatty acid oxidation (FAO) for energy production, and (iii) serve as precursors for the synthesis of signaling molecules such as prostaglandins, thromboxanes, and lysophosphatidic acid [45–48] (Fig. 1).

At variance with normal cells, which derive their lipids from dietary intake or from lipid metabolism in the liver, cancer cells activate de novo lipogenesis, whose limiting enzyme is the fatty acid synthase (FASN, Fig. 1). FASN is overexpressed in several types of human malignancies, including BC, and is frequently associated with aggressive disease course [49–54]. Other enzymes in the de novo FA synthetic pathways are also upregulated in cancers [47]. Cancer cells utilize de novo synthesized FAs for the biochemical functions listed above, including the production of energy through FAO that, under conditions of environmental stress and/or glucose deprivation, is upregulated through overexpression of the critical enzymes CPT1 and CPT2 and becomes the major source of energy for certain tumors [55–58] (Fig. 1).

In addition to de novo lipid synthesis, cancer cells can also increase FA uptake from the extracellular interstitium through several plasma membrane proteins, including the LDL receptor and the FA translocase CD36, whose upregulation associates with poor prognosis in several types of cancers [59–62] (Fig. 1).

The metabolic alterations of cancer cells have long been considered as (quasi)cancer-specific, hence the general definition of “metabolic reprogramming” applied to cancer metabolism. Metabolic reprogramming can indeed occur in cancer due to mutations in metabolic relevant genes, such as isocitrate dehydrogenases (IDH1 and IDH2) in acute myelogenous leukemia (AML) and glioblastoma [63–65] (see later in Fig. 4). This, however, seems be the exception rather than the rule. More frequently, metabolic alterations in cancer are consequent to the subversion of major oncogenic and/or tumor suppressor pathways, including RAS, p53, MYC, PI3K, and mTOR, which can profoundly alter energy production, biomass synthesis, and redox control. While we refer the reader to insightful reviews on this specific topic [66, 67], we would like to point out a couple of important features: (i) the wide mutational variability of cancers appears to converge on a limited number of metabolic phenotypes, although a word of caution might be in order here as less studied metabolic routes are also emerging in cancer metabolism, together with the discovery of novel oncometabolites [68]; (ii) there is no biunivocal correspondence between mutational variations and metabolic phenotypes. Indeed, the same genetic alteration can induce different metabolic phenotypes as a function of context, e.g., cell of origin, architecture of the host tissue, epigenetic context of the cell of origin and of the surrounding normal cells, etc. [69–72].

The emerging picture is that of cancer cells activating and/or coopting metabolic programs present also in physiological conditions, rather than reprogramming their own metabolism in novel aberrant fashions. For instance, the Warburg effect, is utilized by normal cells—neurons, endothelial cells, monocytes, neural crest cells, pluripotent stem cells, and presomitic mesoderm—to execute physiological functions [73–81]. Even in isogenic cancer populations, such as cells in culture, variations in the glycolytic rate are present [82, 83]. Furthermore, dependence on amino acid metabolism is not a specific feature of cancer cells, as it accounts for most of the biomass in all proliferating cells, even normal ones [84]. Thus, an intrinsic plasticity of metabolism itself, rather than a strictly deterministic mutationally driven reprogramming, might be at the core of the metabolic alterations detected in cancer.

### The tumor microenvironment (TME) viewpoint

It has been long recognized that the TME plays a crucial role in the initiation and progression of cancer in general [85], and of BC in particular [86–88]. Before entering in the analysis of the molecular and metabolic circuitries underlying the network of interactions established within the TME, we would like to discuss how the global vision of the TME, afforded by omics approaches, is changing our ability to predict cancer aggressiveness and therapeutic decisions. We will use BC as a paradigm.

The characteristics of the TME play an important role in determining cancer aggressiveness in BC, in addition to the cell-intrinsic features of the various molecular subtypes [89]. For instance, relative to the immune component of the TME, in The Cancer Genome Atlas (TCGA), six immune BC subtypes could be distinguished: wound healing, IFN-γ dominant, inflammatory, lymphocyte depleted, immunologically quiet, and TGF-β dominant [90]. Luminal A BCs are enriched in the wound healing subtype, whereas BCs with high mutational burden display predominantly an IFN-γ dominant phenotype [90]. In another study, transcriptomic analysis of TNBCs revealed different immune (and stromal) profiles associated with overall survival [91]. The advent of single-cell analysis has further corroborated the idea that different immune-related landscapes allow ecologically based stratification of BCs, which, when integrated with the more traditional genetically based classifications, improves prognostic predictions [92–96].

A further step forward relies on highly multiplexed imaging of tumor tissues [97–100], which can be orthogonally coupled to other high-throughput technologies, such as transcriptomics: an approach that is starting to elucidate the complex composition of the BC microenvironment [101, 102]. Danenberg et al*.* have recently systematically mapped TME structures in a large BC cohort and identified ten prototypical structures varying for level of vascularization, stromal activation, and leukocyte composition [103]. Cross-correlation of these structures with genomic data revealed preferential association with BC molecular subtypes and clinical outcome. The structure associated with worst prognosis was the one named “suppressed expansion” in which there was an abundance of suppressive T_reg_ cells and dysfunctional effector T cells, underscoring the impact of the immune microenvironment on the natural history of BC [103].

The variations in TME structure are useful also in the therapeutic setting to predict response. A recent study integrated clinical, digital pathology, genomic, and transcriptomic data collected before treatment in a neoadjuvant setting [104]. Therapy response was determined to a greater degree by the baseline conditions of the entire tumor ecosystem than by the characteristics of the cancer cell component alone [104]. In a different approach, Krug et al*.* profiled, by proteogenomics (mass spectrometry-based proteomics integrated with next-generation DNA and RNA sequencing profiles), a cohort of treatment-naïve BCs in which post-translational modifications had been preserved. The authors found that proteogenomics provided valuable clinical information: (i) it performed better than standard clinical grade technologies for the diagnosis of HER2 + BCs, (ii) it defined subgroups of Luminal A and B tumors with a higher predicted response to immunotherapy, (iii) it improved the prediction of responsiveness to CDK4/6 inhibitors in a subset of TNBCs, and (iv) it identified potential metabolic vulnerabilities [105].

**Fig. 2:**
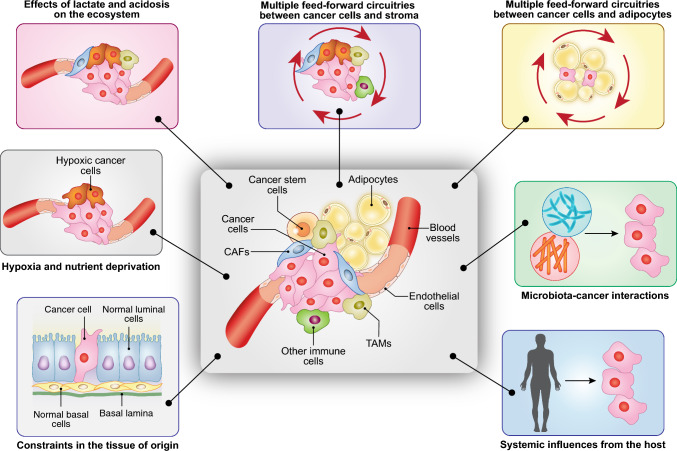
The cancer ecosystem. In addition to cancer cells and cancer stem cells a number of environmental components determines the evolvability of a cancer. From left to right: (i) constraints in the tissue of origin including normal tissue architecture and resident cell types (visualized in a normal mammary gland); (ii) deprivation of oxygen and nutrients, due to the mismatch between cancer growth and its vascularization; (iii) induction of acidosis in the microenvironment and secretion of oncometabolites such as lactate; (iv) triggering of feed-forward loops between cancer cells and stromal cells (cancer-mediated “re-education” of stromal cells), including effects on the ECM and metabolic reprogramming; (v) specific interactions between cancer cells and stromal cells, dependent on the nature of the tissue of origin, e.g., adipocytes–cancer interaction in BC; (vi) interaction of cancer cells with the microbiota (long-range effects and local effects); and (vii) systemic influences due to patients’ metabolism and concomitant pathologies (e.g., diabetes, obesity) and from the diet

Returning now to the cell biology/biochemistry level, the challenge is to deconvolute, at high resolution, the circuitries, first and foremost metabolic ones, through which the cancer–stromal interactions are orchestrated. In a cancer ecosystem, essentially all stromal cytotypes—including immune/inflammatory cells, fibroblasts, endothelial cells, and adipocytes—participate in the modulation of the cancer behavior, sometimes with “suppressive” functions but more frequently aiding the tumor. In this latter case, a variety of circuitries are established between cancer and normal cells that work with the modality of feed-forward loops to create a cancer-friendly environment [106]. These circuitries rely on a variety of secreted (or cell-associated) mediators, such as cytokines, growth factors, adipokines, and hormones that frequently modify the metabolism of both cancer and non-transformed cells. The role of the TME is not limited to the interaction between cell types but also involves the participation of the ECM, tissue stiffness, and of architectural constraints in the organ of origin of the tumor (Fig. 2).

Perhaps not surprisingly, the same metabolic alterations detected in cancer cells are also detected in stromal cells that have been re-educated by the physical and functional interactions with cancer cells, including alterations in glycolysis, one-carbon metabolism, amino acid metabolism, TCA cycle, and FA synthesis. Also in the case of the TME, we are not faced with a cancer-specific rewiring, but rather with adaptations of programs normally enacted, in a more rigorously controlled fashion, by normal stromal cells, to suit the needs of the cancer [107–110]. Herein, we will concentrate on some aspects of the metabolic interactions in the TME involving stromal fibroblasts and immune/inflammatory cells, while we refer the reader to several comprehensive reviews for an in-depth coverage of metabolism in the TME [45, 47, 67, 88, 111–116].

The aforementioned conditions of hypoxia and scarcity of nutrients that affect the cancer cells also influence the surrounding stroma leading to a series of reciprocal metabolic adaptations between the tumor and stroma. For instance, the significant uptake of NEAAs (such as alanine, glutamine, serine, and cysteine) from the interstitium by cancer cells leads to their local depletion [117]. Cancer cells cope with this hazard by enacting a variety of strategies, for instance, by activating degradation of the ECM and taking up the products by macropinocytosis [39]. An alternative strategy involves the activation of stromal cells in the TME [112, 116]. In pancreatic cancer, it has been shown that cancer cells activate a stromal population, the pancreatic stellate cells, to induce their autophagy and the release of alanine which is, in turn, captured by cancer cells to sustain their metabolic needs [43].

The consumption of glucose by the cancer epithelial component leads to glucose depletion in the TME and to the decrease of the ATP:AMP ratio in the stromal fibroblasts (cancer-associated fibroblasts, CAFs). This, together with hypoxia and cancer-derived ROS, trigger the already mentioned reverse Warburg effect in CAFs, leading to increased glycolysis and lactate release that can be used by the cancer cell as a high-energy carbon source [118, 119]. The role of lactate in the cancer-oriented economy of the TME is multifaceted (see also section A1). Lactate stimulates neo-angiogenesis of the tumor [120–122] and affects the quality and activity of the immune infiltrate in the TME. In general, lactic acid dampens the activation of cytotoxic T cells and natural killer cells, while leaving immune-suppressive cells (e.g., T_reg_ cells) unaffected or even stimulating them. Furthermore, lactate can induce the polarization of TME macrophages toward an M2-like phenotype which is associated with an anti-inflammatory action and poor prognosis in BC. Finally, lactate can interfere with the activity of antigen-presenting cells. The combination of these effects strongly contributes to tumor immune evasion [27–29, 115].

## The BC metabolic ecosystem

In this section, we describe the evolution of the BC metabolic ecosystem at various stages of the disease, from the moment of the diagnosis and the transition from in situ to invasive cancer to the acquisition of the metastatic phenotype and the dormancy/reactivation of the metastatic cell.

### Molecular, phenotypic, and metabolic heterogeneity in BC

The molecular heterogeneity of the different BC subtypes is associated with distinct metabolic features [123, 124]. For instance, there are substantial differences in glutamine dependence, with basal (TNBC) tumors being in general more glutamine-addicted than Luminal tumors [125]. This is, in some cases, due to the preferential activation of the MYC or JUN oncogenes in TNBC, which leads to increased dependency on glutamine metabolism and uptake [126–128].

Serine metabolism is also differentially altered. 3-phosphoglycerate dehydrogenase (PHGDH), the first enzyme involved in serine synthesis, is overexpressed in ER-BCs and promotes aggressive rates of proliferation [37, 129, 130]. In addition, TNBCs depend on FAO to maintain elevated SRC activity, which is in turn needed for metastasis [131].

The FASN gene, involved in de novo FA synthesis, is overexpressed in HER2 + BCs and might represent a therapeutic target in these tumors, as it is also involved in brain metastatization of these tumor subtype [132–134]. Interestingly, the CD36 FA translocase is upregulated during the development of therapy resistance in HER2 + BCs, in a process that shifts the dependency of these tumors from de novo, FASN-dependent, synthesis of FA to uptake of exogenous FAs [135].

In ER + Luminal BCs, the AAMDC oncogene (adipogenesis associated Mth938 domain containing) is amplified and regulates the expression of several metabolic enzymes involved in the one-carbon folate and methionine cycles [136], a feature shared with the CDK12 (cyclin-dependent kinase 12) oncogene [137].

A recently added level of complexity concerns the assembly of multi-enzyme complexes in the glycolytic pathway operated by long non-coding RNAs (lncRNA). One such lncRNA, NEAT1, functions as a scaffold for a complex comprised of three glycolytic enzymes, PGK1 (phosphoglycerate kinase), PGAM1 (phosphoglycerate mutase), and ENO1 (Enolase), which accelerates glycolysis through substrate channeling, a process in which the processed intermediate is transferred from one active enzymatic site to the next, preventing its free diffusion [138]. Ablation of NEAT1 attenuates aggressive BC progression and metastasis in mouse models as a result of reduced glycolysis. NEAT1 is present in two isoforms, produced by alternative 3′ processing: NEAT1_1 and NEAT1_2. Only NEAT1_1 participates in the formation of the PGK1/PGAM1/ENO1 complex [138]. Interestingly, NEAT1_1 is an ER target gene and is preferentially overexpressed in Luminal BCs [139, 140]. How this might relate to dependence of Luminal BCs on increased glycolytic flux or to aerobic glycolysis is not known.

In conclusion, while the picture is still blurry, it appears that the different molecular subtypes of BC are also metabolically distinct.

A second level of variability is provided by metabolic heterogeneity within BC subgroups. For instance, in Luminal B BCs, as compared to Luminal A, there are significant differences in the glutamine–proline regulatory axis, evidenced by high levels of glutaminase (GLS), pyrroline-5-carboxylate synthetase (ALDH18A1), and pyrroline-5-carboxylate reductase 1 (PYCR1) [141]. However, most efforts in this area of research have been directed at the identification of metabolic variability and vulnerabilities within the TNBC subtype, given the pressing need for novel therapeutic targets in this poor-prognosis, relatively therapy refractory, BC subtype. Multiomics analysis has led to the identification of distinct metabolic alterations within TNBCs [142], allowing, for instance, their subclassification into lipogenic, glycolytic, and mixed subtypes [143]. Importantly, these potential differential vulnerabilities correspond to increased sensitivity to specific metabolic inhibitors and, in the case of glycolytic tumors, to synergistic effects in response to combined anti-metabolic and immune therapies [143].

Overall, the emerging picture highlights that while several subverted metabolic phenotypes are evident within BC subtypes, there is also a remarkable level of intra-subtype heterogeneity. It remains to be established, how much of the intra-subtype variability can be ascribed to cancer cell-autonomous events *vs*. the more complex scenario of different cancer ecosystems generated by the interaction of subtly different starting microenvironments interacting with cancer cells. In general, the results also point to an intrinsic difficulty of using a purely subtype-based approach to identify metabolic vulnerabilities in BC.

Some of these problems might be resolved by the application of methodologies affording single-cell resolution of the cancer and non-cancer components [144, 145]. These technologies—including single-cell sequencing, mass cytometry, multiplexed imaging, and spatial transcriptomics—are being applied to the study of BC revealing a staggering level of intra-tumoral heterogeneity, which we will now discuss.

### Intra-tumoral metabolic heterogeneity in BCs

Pioneering work, based on microdissection of cancer areas from the same tumor or on deep-sequencing followed by deconvolution of the clonal evolution of individual tumors, initially identified intra-tumoral molecular heterogeneity across various BC subtypes [146–148]. The advent of high-resolution technologies for single-cell analysis has subsequently solidified this concept, further establishing that both genetic and epigenetic alterations contribute to intra-tumoral heterogeneity [149–153]. Not surprisingly, clonal evolution of tumors, and the ensuing intra-tumoral heterogeneity, is a multi-dimensional process, in space and time [93, 94, 148, 150, 154, 155]. Recent analyses, based on multiplexed mass cytometry, further demonstrated that vastly heterogeneous cellular communities of cancer and stromal cells co-exist in the same primary tumor, harboring relevant clinical information toward disease stratification and clinical outcome [101, 102]. A detailed analysis of this topic is beyond the scope of this review; thus, we refer the reader to recent comprehensive reviews [144, 156, 157]. Rather, we will discuss how much of this intra-tumoral molecular heterogeneity translates into intra-tumoral cancer cell-specific metabolic heterogeneity in BC.

Here, the evidence is still sparse. Singh et al*.* showed that overexpression of PHGDH, which is involved in serine synthesis and promotes aggressive behavior in TNBCs, is heterogeneous in TNBC cell lines [158]. The heterogeneous expression of PHGDH might be due, at least in part, to metabolic stresses from architectural constraints, underscoring the possible relevance of microenvironmental components to metabolic heterogeneity [158]. The case of PHGDH is a puzzling one, as it was recently reported that loss of PHGDH expression potentiates metastatic dissemination in animal models and is associated with decreased metastasis-free survival time in BC patients [159]. Interestingly, the pro-metastatic effect is not due to loss of the enzymatic function of PHGDH, but rather to loss of a non-enzymatic function involving a non-catalytic interaction with the glycolytic enzyme phosphofructokinase leading to aberrant protein glycosylation through activation of the hexosamine–sialic acid pathway. In a nutshell, high PHGDH promotes tumor growth, while low PHGDH promotes metastasis. A possible reconciliation, advanced by the authors, is that amplification/overexpression of PHGDH might put the cell into a sort of metastable state, in which regulation of PHGDH levels might determine the pro-tumorigenic *vs*. pro-metastatic states [159]. It must also be pointed out that the existence of non-catalytic functions of bona fide metabolic enzymes represents an unappreciated level of complication to account for.

Another documented case of intra-tumoral heterogeneity concerns Luminal BCs. The Luminal BC cell line MCF7 displays uniformly a mutation of the PIK3CA gene, known to stimulate increased glycolysis [160]. Yet, within the cell line, the uptake of glucose and the rate of glycolysis are not uniform [83]. Also in this case, architectural components, depending on cell density, might drive the intercellular heterogeneity.

Some of these studies suffer of some intrinsic limitations. First, in some cases, results were obtained in cell lines, which are not necessarily faithful representations of the epithelial component of BCs. Second, while cell lines might be useful in isolating the cell-autonomous component from the non-cell-autonomous one, the complexity of the cancer ecosystem is lost in this setting, although—as discussed—some architectural components can still be unveiled. It is not going to be easy to transfer these results to the complexity of real cancers. One major difficulty is that single-cell analysis techniques, available for analysis on tumor specimens, provide measurements of transcripts and, sometimes, proteins, but no direct analysis of metabolites. Thus, in the best case, metabolic alterations must be inferred from patterns of expression, but not from direct measurements. A possible solution to circumvent some of these problems is to employ mouse models of BC. In one such study, it was shown that individual tumors are intrinsically metabolically heterogeneous, displaying both glycolytic and OXPHOS cells [161]. Efforts have also been directed at the creation of modeling frameworks in which gene regulation datasets are coupled to actual metabolic pathway measurements [162].

In addition to genetic variations, one of the most prominent aspects of intra-tumoral metabolic heterogeneity relates to the spatial distribution of cancer cells within the tumor mass, which differentially affects their accessibility to oxygen and nutrients. Imperfect nutrient and oxygen distribution throughout tumors is a major confounding factor when comparing clonal evolution, epigenetic imprinting, and other more stable alterations because this metabolic tumor zonation impacts on epigenetic remodeling and clonal selection driving large part of the tumor evolution process. To investigate this aspect, a wealth of new applications, exploiting orthogonal measurements including metabolic ones, is being developed for applications in vivo and ex vivo and might help in the deconvolution of spatial and temporal organization of metabolic programs in several cancers, including BCs [163–167].

### Metabolic phenotypes in the natural history of BC: the transition from in situ to invasive cancers

**Fig. 3:**
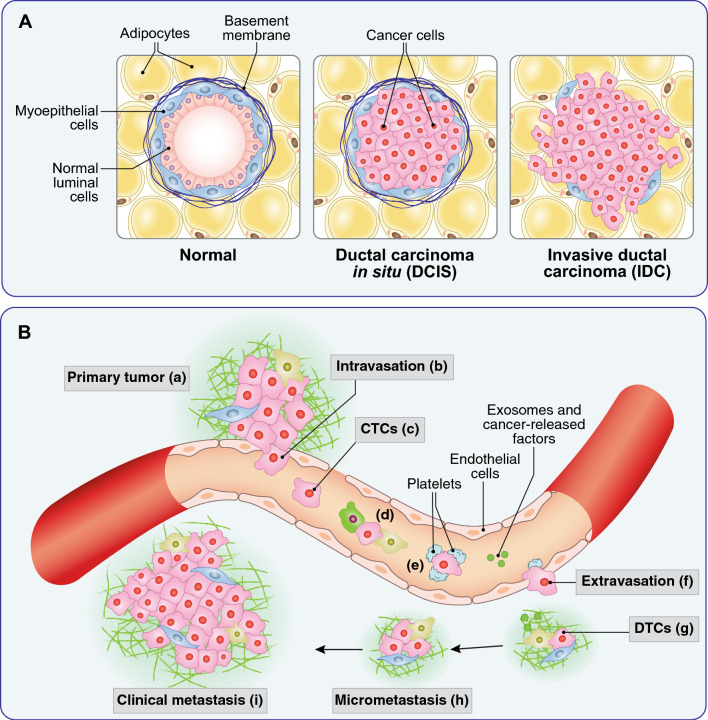
BC invasiveness and metastasis. **A** From DCIS to IDC. In the normal breast gland (left), two ordered layers of luminal and myoepithelial cells are present, separated from the surrounding stroma by a basement membrane. In DCIS (center) cancer cells proliferate and fill the lumen, while remaining confined by an intact myoepithelial–basement membrane barrier. In IDC (right), cancer cells penetrate into the surrounding stroma, with loss of the basement membrane and of the myoepithelial barrier. **B** Metastasis as a multistep process. Locally invasive cancer cells from the primary tumor (a) are able to intravasate into the local circulation (b) also because of the poor structure of the neo-formed tumor vessels that present fenestrations with interruption of the endothelial and pericyte (not shown) layers. Once in the bloodstream, cancer cells are defined circulating tumor cells (CTC, c). In the bloodstream, CTCs need to survive shear stress and predation by immune cells (d), which they do also with the aid of a platelet coat (e). In distant organs, CTCs attach to endothelial cells and extravasate (f) becoming disseminated tumor cells (DTCs, g). The settlement in the distant organs (micrometastasis, h) is facilitated by cancer-released exosomes and factors (g, green dots) that prepare the so-called pre-metastatic niche. Micrometastases can remain dormant for long periods of time, before being reactivated and giving raise to macrometastases or clinically detectable metastases (i). Each single step of the metastatic process is accompanied to distinct metabolic changes, reviewed extensively in [170]

DCIS is a pre-invasive malignant form of BC characterized by the separation of cancer cells from the surrounding stroma by a near continuous layer of myoepithelial cells which supposedly represents a barrier to infiltration into the adjacent stroma (Fig. 3). While the condition is not life-threatening per se, around 50% of DCIS patients will develop invasive ductal cancer (IDC) [168]. DCIS lesions, which are increasingly being detected through BC screening programs, represent, therefore, a unique opportunity to study early phases of the BC natural history and the molecular and phenotypic events involved in disease progression. Thus, concerted investigations have been directed at studying genomic alterations involved in the DCIS-to-IDC transition [150, 169]. By comparing DCIS and their matching IDCs, it was revealed that most genetic alterations evolved in the duct prior to invasion, compatible with a multiclonal invasion model with no specific advantage-conferring genetic event responsible for the invasion.

Because of these findings, efforts have been directed at deciphering changes in the TME that correlate with the acquisition of invasive phenotypes in DCIS. The use of multiplexed ion beam imaging by time of flight (MIBI-TOF) [171] has been instrumental to this end. MIBI-TOF—coupled with multiplexed antibody and correlation with transcriptomics data, cellular composition, and structural characteristics of the normal tissue—was used to characterize DCIS and IDC from the same patients [172]. The transition from DCIS to IDC appeared to be characterized by coordinated stages of TME alteration, as evidenced by the location and function of fibroblasts, immune cells, and myoepithelial cells. Surprisingly, it was found that progressing DCIS was characterized by lower levels of myoepithelial disruption compared with non-progressing DCIS. These results are consistent with the counterintuitive notion that a compromised myoepithelial barrier facilitates sensing of the tumor by other stromal components providing protection against invasiveness and progression [172].

In another approach, Lomakin et al*.* developed BaSSIS, a highly multiplexed fluorescence microscopy-based pipeline, to address the topology and phenotypic characterization of subclones in individual cancers, and applied it to the study of BC progression (DCIS to IDC to lymph node metastasis) [173]. In the case of DCIS, while the macroscopic scale was dominated by polyclonal expansions, individual clones segregated into microanatomical structures, characterized by distinct transcriptional and histological features and cellular microenvironments.

These studies are starting to provide a spatial atlas of BC progression and direct evidence, in real tumors, that studying cancers as individual ecosystems, rather than assembly of cellular subsets, might change our understanding of the natural history of the disease. The metabolic question then is as follows: does metabolic plasticity contribute to the establishment/maintenance of subclonal territories, and does this impact the natural history of BC? Initial answers are being obtained. In DCIS, the peculiar modality of growth (inside the lumen, progressively further away from blood supply) can generate harsh conditions of nutrient deprivation, which might promote the onset of aerobic glycolysis. In vitro, BC cell lines subjected to nutrient deprivation indeed develop a Warburg phenotype, probably due to the induction of the transcription factors KLF4 and NFκB. In actual DCIS, KLF4 is enriched in areas of harsher microenvironmental conditions, especially closer to the necrotic core of the lesions [174].

**Fig. 4:**
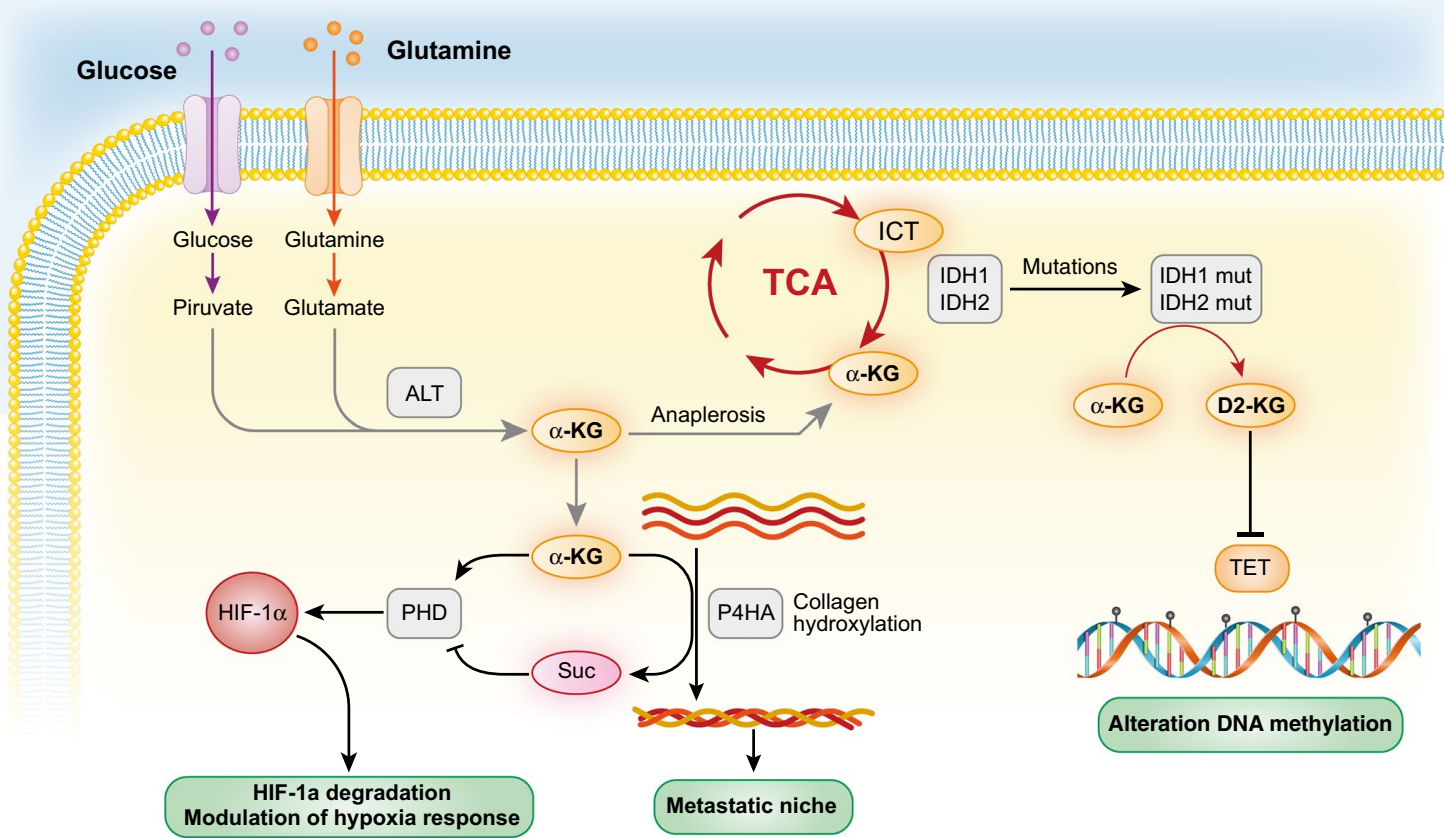
α-KG and its alterations in cancer. The figure depicts a number of metabolic alterations centered on α-KG in cancer. The metabolism of α-KG is not exhaustively represented and a more detailed description can be found in [181]. The cancer phenotypes (in green boxes) dependent on α-KG and on its metabolite D-2HG are described in this section and in section E, respectively. Abbreviations are spelled out in the main text

An interesting connection between metabolism and invasiveness stems from studies of collagen metabolism and of prolyl-4-hydroxylase (P4HA), the enzyme that promotes proline hydroxylation of collagen and the formation of the collagen triple helix. It is known that collagen production, mostly by stromal cells, is required for BC progression [175]. Interestingly, however, P4HA is overexpressed in TNBC and HER2 + BCs and is required for invasiveness, through increased secretion of collagen by cancer cells, correlating with poor clinical outcome [176, 177]. The effects of P4HA overexpression in some BCs might be further enhanced by pyruvate, which is converted in BC cells into α-KG (through the alanine aminotransferase reaction which converts pyruvate and glutamate into α-KG and alanine), a cofactor of the collagen hydroxylation reaction by P4HA [178]. This is, in turn, needed for the collagen-based remodeling of the metastatic niche by BC cells [178]. While it is not clear what the source of pyruvate might be in the TME, it is worth noting that lactate, an abundant oncometabolite in the microenvironment, can be taken up by cancer cells and converted into pyruvate [179]. The impact of P4HA levels/activity on BC progression might extend beyond its role in collagen remodeling. In the collagen hydroxylation reaction, P4HA consumes α-KG, leading to accumulation of succinate. The regulation of these two oncometabolites impacts on hypoxia response. Indeed, α-KG is also a cofactor for PHD enzymes (oxygen-dependent dioxygenases) which hydroxylate HIF-1α (a subunit of the HIF-1 transcriptional complex), leading to its ubiquitination and degradation [180]. Thus, the consumption of α-KG by P4HA and the accumulation of succinate, which inhibits PHDs, leads to stabilization of HIF-1α and increased hypoxia response: this was shown to correlate with the acquisition of stem cell traits and metastatic ability by BC cells [176, 177] (Fig. 4).

Finally, it has been shown that BC cells, at variance with normal mammary cells, depend on glutaminolysis. The product of this reaction is glutamate, which in BC cells, but not in normal cells, can be extruded from the cell by the xCT antiporter system. Since the overexpression of the xCT antiporter system correlates with BC aggressiveness and poor prognosis [127, 182], it was investigated whether glutamate extrusion and extracellular accumulation was responsible for these effects. Indeed, excess glutamate activates on the surface of BC cells, likely through a paracrine effect, the metabotropic glutamate receptor GRM3 that, in turn, triggers recycling of the protease MT1-MMP, via a Rab27-dependent pathway, to active invadopodia, thereby allowing matrix degradation and invasion [183]. It is worth mentioning that excess glutamate might also foster the ALT (alanine transaminase) reaction, which leads to increased α-KG levels, linking together the complex network of metabolic alterations described (Fig. 4).

### Metabolic phenotypes in the natural history of BC: the determination of the metastatic phenotype

**Fig. 5:**
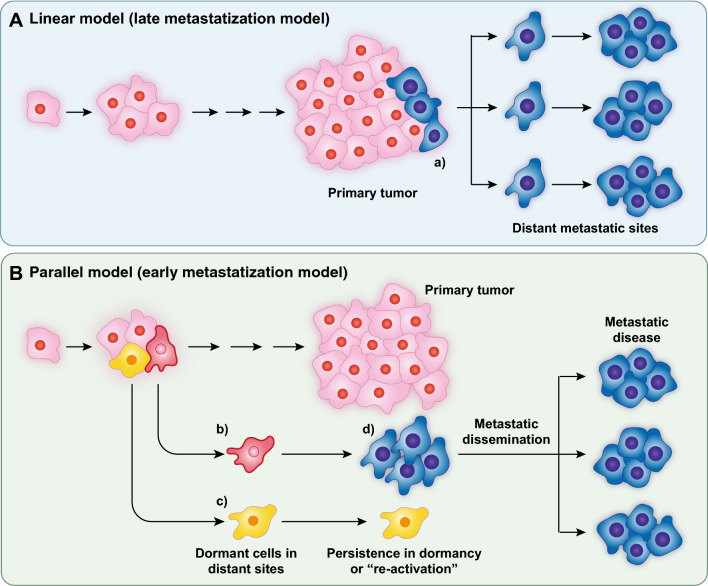
Models of metastasis. **A** In the linear model of metastasis a clone of cells, genetically endowed with metastatic potential (a, in blue) develops late in the natural history of the tumor and gives raise to metastases. **B** In the parallel model of metastasis, metastatic cells (b and c, in red and yellow) detach early from the primary tumor, reach the peripheral organs, and might undergo long periods of dormancy. These cells are probably not genetically altered, but rather underwent a process of P-EMT under the influence of metabolic alterations. Most of the metastatic cells (exemplified here by the cell c) will never exit dormancy. In some cases, a dormant cell (d, in blue) might be reactivated and rapidly expand giving raise to multiple metastases in several organs (metastatic disease). The mechanisms of reactivation are poorly characterized and there is evidence that they depend both on genetic and non-genetic (including metabolic) alterations. The scheme in B is simplified and depicts metastases as monoclonal; there is evidence that some metastases might be polyclonal, probably due to collective migration of cells from the primary tumor. The majority of evidence, however, supports a monoclonal model, especially for the clinically evident metastases (reviewed in [193])

The pioneering experiment of Fidler and Kripke showed that highly metastatic cell variants preexisted in a parental population of cultured melanoma cells [184]. This led to the linear (or late) model of metastatization in which metastases were considered a late, genetically determined, event in the natural history of the tumor (Fig. 5A). In the case of BC, mounting evidence supports a different model, in which metastatic spreading is a very early event [185–187] (Fig. 5B). In addition, molecular evidence argues against the concept of genetically driven selection of the metastatic phenotype. Indeed, several studies have shown that driver mutations and gene copy number variations mostly overlap in primary tumors and their synchronous metastases [188–192].

These findings raise the question of what determines the onset of the metastatic ability in the primary tumor. There is evidence that this phenotype represents an adaptive response to environmental hazards, including hypoxia, nutrient deprivation, and the ensuing metabolic stress [194]. The enacted cellular responses appear to converge on the induction of EMT. Here, we provide a brief summary of the current knowledge on EMT, for a more in-depth review see [195].

EMT is a program that allows epithelial sessile cells to switch to a migratory mesenchymal-like state. In the physiological setting, EMT is highly relevant during morphogenesis [195, 196]. In cancer, EMT is connected to the acquisition of invasive/metastatic ability, cancer stem cell-like properties, and of therapy resistance [197–201]. EMT is best viewed as a series of interconvertible metastable plastic states that yield cells displaying full EMT or cells with intermediate (and reversible) phenotypes (P-EMT: plastic, or partial, EMT). In this latter state, context and metabolism are emerging as major determinants [197, 202–207].

In BC, it was shown that hypoxia enhances EMT and correlates with metastatic progression and poorer patient outcome [13–15]. In response to hypoxia, cancer cells stabilize HIF-1α, which, in turn, regulates transcription of several target genes, including transcriptional regulators of EMT (SNAIL, ZEB1 and TWIST), glucose transporters, glycolysis enzymes, and VEGF [13–15]. Accordingly, negative modulation of HIF-1α inhibits EMT and metastatic progression in BC [208, 209]. Hypoxia might exert pleiotropic effects on the determination of the metastatic phenotype. It was shown that hypoxia-induced expression of the transcriptional coactivator PGC-1α (a.k.a. PPARGC1A) enhances mitochondrial biogenesis and oxidative phosphorylation and promotes the formation of distant metastases. Interestingly, the induction of EMT and PGC-1α was independently regulated by hypoxia, suggesting synergistic effects on the metastatic phenotype [210].

Another study highlighted a novel connection between hypoxia and EMT, as it was shown that BC cells release extracellular vesicles that can reprogram mitochondrial dynamics and function and induce EMT once seized by normal mammary cells [211]. It is not clear whether and how the induced modification of normal mammary cells can contribute to the BC ecosystem. One possibility is that it might contribute to a “field effect” of cancerization: a process by which genetic and epigenetic changes are induced by cancer (or pre-cancerous) cells in the normal adjacent epithelia, causing the replacement of the normal population with a cancer primed population (see [212] for a review of the concept). It is also reasonable to imagine that extracellular vesicles released by hypoxic cancer cells might induce a metabolically altered phenotype, and EMT, in other cancer cells not exposed to the hypoxic hazard, thereby amplifying the metabolically altered territory.

Nutrient depletion might represent another hazard to which BC cells respond with metabolic adaptation, leading to EMT and increased metastatic ability. Glutamine deprivation of BC cells activates the expression of stress response and pro-inflammatory genes [213]. Mechanistically, this was linked to EMT and metastasis through the demonstration that glutamine-deprived cells upregulate asparagine synthetase (ASNS), thus becoming asparagine addicted [214]. In turn, ASNS stimulates EMT of BC cells and their migration/metastasis [215].

Another link between the metabolic state and EMT comes from a multiomics analysis of 180 cancer cell lines [216]. In this study, there was a strong association between individual cellular metabolomes and EMT transcriptional signatures, and evidence was provided that metabolic alterations might act upstream of EMT.

The sum of the evidence suggests that responses to metabolic stresses induce migratory ability, through EMT, thereby allowing cancer cells to remove themselves from areas of high hazard to access areas of improved resource availability within the primary tumors. Since these areas are typically in the immediate periphery of leaky and highly permeable vessels, the extravasation and dissemination of cancer cells might, therefore, represent a non-selected consequence of their increased EMT-dependent migratory activity. In support of this possibility, it should be remembered that EMT is also inextricably linked to the cancer stem-like state and that the metastatic ability of BCs (and the prognostic outcome) is a direct function of the number of cancer stem cells present in the primary tumor [217, 218].

### Metabolic phenotypes in the natural history of BC: exosomes and the pre-metastatic niche

Exosomes are a subset of extracellular vesicles, released by cells, which can deliver their content to adjacent cells [219, 220]. The ability of exosomes to carry lipids, proteins, and nucleic acid allows for efficient intercellular communication. In particular, the delivery of nucleic acids, first and foremost miRNAs, enables exosome-donating cells to reprogram (epi)genetically exosome-receiving cells [219, 220]. In BC or in BC models, bi-directional exchange of exosomes between normal and cancer cells (reviewed in [221, 222]) plays a role in the primary tumor [223], in the development of resistance to therapy [224], in the establishment of successful metastatic colonization [225–227], and in the preparation of the so-called pre-metastatic niche (PMN) (see also Fig. 3). This latter structure is of particular interest. PMNs are established in target organs of future metastasis through the concerted action of soluble factors and exosomes released from the primary tumors and capable of long-distance action [228]. PMNs differ substantially from metastatic niches in that the latter are established by disseminating tumor cells (DTCs) upon their arrival, while PMNs are devoid of tumor cells and represent a tumor-favorable environment established at a distance by the primary tumor. The modifications induced in the PMN include development of inflammation, angiogenesis, and of an immune-suppressive environment [228].

In a landmark study, Hoshino et al*.* demonstrated that various integrins expressed on the surface of exosomes released from BC cells can direct the exosomes to specific organs, delivering their cargoes to various normal target cells and activating pro-inflammatory programs. This exosomal-mediated reprogramming could be correlated with clinical data, since, in BC patients, the specific integrin exosomal patterns correlated with the site of metastasis [229]. The metabolic relevance of exosomal-mediated reprogramming for the establishment of the PMN was established by showing that exosomes, secreted by BC cells, are loaded with miR-122. This miRNA is capable of suppressing glucose metabolism in target cells by downregulating the production of the enzyme pyruvate kinase. In animal model systems, the injection of miR-122-containing exosomes reduced glucose consumption in brain and lung and was associated with increased colonization of these organs by injected BC cells [230]. Thus, exosomes might prepare the PMN by reducing glucose consumption by resident cells, to make more resources available to DTCs upon their arrival.

The impacts of exosome delivery to PMNs might be complex. In the case of lung cancer, for instance, it was shown that tumor-derived exosomes can re-educate resident macrophages toward an immunosuppressive phenotype through increased expression of the immunosuppressant molecule PD-L1, downstream of the activation of the Toll-like receptor TLR2 [231]. Mechanistically, it was shown that the upregulation of PD-L1 was due to metabolic reprogramming, through upregulation of glycolysis [231]. Apparently, therefore, we are faced with the contradiction that exosomes prepare the PMN both through reduction of glycolysis [230] or its increase [231]. Cancer-specific differences (breast *vs.* lung) might be responsible for these opposing results. Alternatively, it is tempting to speculate that the complement of molecules exposed on the surface of endosomes might direct them to different cellular targets in the PMN, thereby allowing selective modulation of metabolism in different types of organ-resident cells.

Another interesting question concerns whether the exosome-instructed modulation of the PMN is a selected event during the natural history of the tumor. From an evolutionist viewpoint it is difficult to imagine that a phenotype is selected if it does not provide an immediate advantage. One should ask whether exosome secretion confers advantages in the primary tumor, with the long-distance effect on the PMNs being an unfavorable (for the patient) accident. In this context, it is interesting to note that overexpression of miR-122 reduces the growth of primary tumors, while fostering that of metastases [230]. Thus, it is possible that decreasing miR-122 in the primary tumor, through its exosomal secretion, might represent an advantage-conferring event.

Finally, a different facet of the active role of metabolism in the establishment of the PMN has emerged from evidence that nutrients available in distant organs may promote metastatic growth. In the lung, the PMN is enriched in palmitate and promotes metastatic growth of BC cells. These cells use palmitate to synthesize acetyl-CoA which is exploited to increase protein acetylation resulting in pro-metastatic NFκB signaling [232].

### Metabolic phenotypes in the natural history of BC: dormancy

Migration out of the primary tumor represents only the initial phase of the metastatic journey, in which DTCs must face a series of hurdles, including survival in the blood stream, extravasation, and implant/survival/proliferation in distant organs. While we will not dwell on a detailed description of these phases (for a review see [233]), it is important to note that the process is highly inefficient. It has been estimated that in BC, only 0.01% of the cells that manage to enter the blood stream will eventually form metastases [234]. A peculiar characteristic of BCs is that a significant fraction of patients develops metastatic disease after years or even decades of dormancy after the primary tumor was successfully cured (Fig. 5). This implies that a number of DTCs (or of subclinical micrometastases) were present at the time of diagnosis and that these cells remained dormant for long periods of time [235–237]. The phenomenon of dormancy raises questions of obvious relevance to the understanding of the metastatic process and to the clinical management of cancer patients. What determines dormancy? How do DTCs eventually exit from dormancy? How is the survival of DTCs ensured during dormancy?

Answers to these questions are limited. Dormancy appears to be largely mediated by interactions of DTCs/micrometastases with the growth suppressive environment of newly colonized tissues. A number of negative cues, derived from the microenvironment—including the perivascular niche, immune/inflammatory cells and other cell types—promotes dormancy ([238–240], reviewed in [236]). Conversely, the exit from dormancy and the development of clinically detectable metastatic disease might be relevantly impacted by genetics. Indeed, while the genetic landscapes of primary BCs and of their synchronous metastases are largely overlapping [188–192], the situation could be different when metastases arise years after the removal of the primary tumor (metachronous metastases). In this case, the genetic landscape is significantly altered *vs*. the primary tumor, and metastases seem to have accumulated independent mutations (drivers and passengers) in late phases of their development [241, 242]. Thus, a scenario can be envisioned in which the initial metastatic dissemination is an early event in the natural history of BCs, driven essentially by metabolic adaptation to a harsh environment. DTCs are then induced to dormancy by inhibitory cues derived from the microenvironment of the newly colonized tissues. Finally, in some cases, dormant metastases can be reactivated by new fitness-increasing genetic alterations, which can give rise to the onset of the clinical metastatic disease (Fig. 5), compatible with a model of “metastatic horizontal self-seeding” in BC and in other types of cancer [243, 244]. It must be noted, however, that the tissue microenvironment has an impact also in this phase of reactivation of dormant metastases as will be discussed in the next section.

The largest gap in knowledge here concerns the long period of dormancy, which can last years: how do dormant DTCs survive in a growth suppressive environment? The question is relevant because it might point to vulnerabilities that can be exploited for therapeutic purposes. While we refer the reader to specific reviews on the metabolism of dormant DTCs [245, 246], we would like to discuss in some detail the emerging evidence pointing to a crucial role of autophagy.

**Fig. 6:**
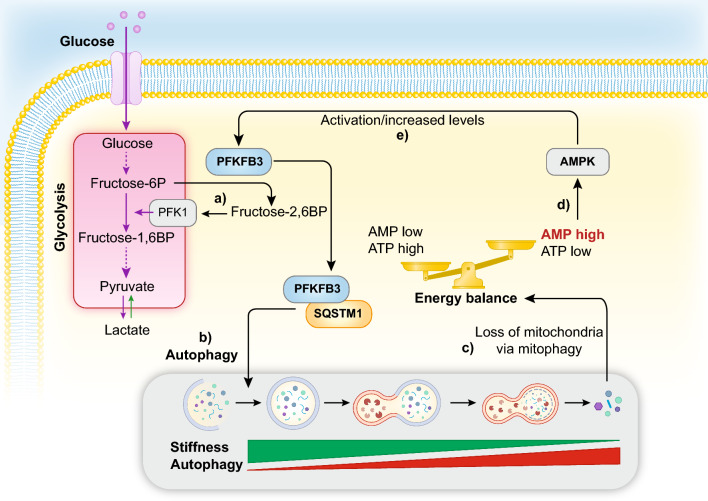
Autophagy, dormancy, and the role of PFKFB3. PFKFB3 converts fructose-6-phosphate (Fructose-6P) into fructose-2,6-bis-phosphate (Fructose-2,6BP), in turn a potent allosteric activator of PFK1 (6-phosphofructokinase-1), thereby stimulating glycolysis (a). In dormant metastatic cells, autophagy sustains survival showing inverse correlation with matrix stiffness. Under conditions of sustained autophagic flux, PFKFB3 is continuously destined to autophagic degradation, through interaction with SQSTM1 (b), thereby attenuating the glycolytic metabolism. Excessive or persistent autophagy, however, can alter the energy balance through mitophagy-dependent loss of mitochondria (c). This, in turn, alters the AMP:ATP ratio in the cell and it is sensed by AMPK (d). AMPK activates PFKFB3 through direct phosphorylation and translational activation of its mRNA (e), thereby restoring high glycolytic flux, associated with exit from dormancy

Autophagy is a conserved cellular recycling mechanism finalized to the removal of misfolded proteins and aged organelles, their targeting to lysosomal degradation, and final re-utilization of the elementary components [247]. From a metabolic point of view, autophagy is a powerful scavenging pathway. While it cannot increase the cell biomass, it is key to maintain metabolic homeostasis in times of limited nutrient availability by digesting intracellular components and also reducing the oxidative stresses through the removal of dysfunctional molecules. Autophagy intersects cancer behavior in numerous and frequently apparently paradoxical manners [248]: for this review, the relevant aspect is that autophagy can sustain the survival of dormant cancer cells (Fig. 6). In xenograft models, it was shown that inhibition of autophagy reduced the survival of DTCs; however, there was little or no effect on metastatic growth once the transition to a non-dormant state was achieved [44]. The protective mechanism likely involves removal of damaged mitochondria and maintenance of redox homeostasis. The microenvironment might play a major role in the entire process, as it was shown, in in vitro systems, that an inverse correlation between matrix stiffness and autophagic activity in BC cells exists [249]. This is interesting since BC metastasis frequently occurs in tissues which are softer than the original mammary gland microenvironment. Thus, tissue mechanics might contribute to the establishment of a dormant state through the activation of the autophagic pathway (for a review on tissue mechanics in cancer see [250]).

Studies of PFKFB3 (6-Phosphofructo-2-Kinase/Fructose-2,6-Biphosphatase 3) provide additional connections between dormancy and autophagy/metabolic status in BCs. PFKFB3 is an enzyme that converts fructose-6-phosphate to fructose-2,6-bis-phosphate, which in turn is a potent activator of 6-phosphofructokinase-1 (PFK-1), thereby stimulating glycolysis [251]. PFKFB3 is upregulated in cancer and its overexpression correlates with poor patient outcome in BC [252, 253]. In addition, PFKFB3 expression and autophagy are inversely correlated; accordingly, dormant BC cells display low PFKFB3 and high autophagy rates, while proliferating BC cells show the opposite phenotype; in agreement, ectopic expression of PFKFB3 allows BC cells to exit dormancy. Mechanistically, autophagy controls the levels of PFKFB3. Indeed, the cargo/autophagic protein SQSTM1 (sequestosome 1) binds to PFKFB3 committing it to autophagic degradation [252]. The scenario might be more complex than this. As a result of prolonged permanence in a dormant/autophagic state, cells can experience mitophagy-dependent loss of mitochondria, accompanied by reduced ATP levels. This activates AMPK, a sensor kinase that activates catabolism in response to stress signals and compromised bioenergetics (through sensing the AMP:ATP ratio) [254]. AMPK activates PFKFB3 through a dual mechanism, direct phosphorylation of the protein, and, possibly, translational activation of its mRNA [255]. Thus, a scenario can be hypothesized in which dormancy is maintained through autophagy and a predominant catabolic state (keeping PFKFB3 suppressed and inhibiting glycolysis) until ATP levels drop below a critical threshold, thereby activating AMP and PFKFB3, resulting in glycolytic shift and exit from dormancy (Fig. 6). Perplexingly, however, AMPK is also known as an inducer of autophagy, with mechanisms different from its control over PFKFB3 [256], and acts as a mediator of BC cell dormancy, at least in ER + BCs [257]. Evidently, much remains to be understood in the regulation of the AMPK–PFKFB3 axis and its involvement in autophagy/dormancy. Of note, PFKFB3 is normally localized in the nucleus, while AMPK is thought to act in the cytoplasm. However, in response to stress, PFKFB3 can be acetylated and retained in the cytoplasm, thus making it available for the phosphorylation by AMPK [258]. Thus, cell-specific or cell state-specific differences in the post-transcriptional modification of PFKFB3 might affect the outcome of the AMPK–PFKFB3 circuitry. It should be finally pointed out that PFKFB3, through its glycolysis-activating function has a major role in endothelial cells in sustaining vessel sprouting [75] and that its inhibition leads to tumor vessel normalization and reduced metastasis [259]. It is not obvious how this is connected to the overexpression of PFKFB3 in epithelial cancer cells. One possibility is exosomal-mediated reprogramming of the microenvironment by cancer cells. Indeed, there is one report (in need of further confirmation) of secretion of PFKFB3 by cancer cells with consequent effects on endothelial cell proliferation in a nasopharyngeal cancer setting [260].

It is finally worth mentioning that there is increasing evidence linking autophagy to the maintenance of stem cell state, pluripotency, and self-renewal [261–264], raising the possibility that dormancy, and its metabolic characteristics, might simply represent a natural extension of the properties of the migrating/metastasizing cells, as discussed above.

### Metabolic phenotypes in the natural history of BC: the “reactivation” of dormant metastases

Exit from the dormancy state probably involves, at least in part, fitness-increasing genetic alterations developed after the initial metastatic event, as discussed above. However, the overall picture is still tentative, as our understanding of the metastatic process is undergoing a major overhauling in recent years. For this review, some relevant questions are as follows: is the tissue microenvironment at the site of metastasis involved? And also is metabolic plasticity involved in the exit from dormancy? Efforts are starting to be directed at answering these questions in BC.

One interesting feature of BC is that while metastases can occur in several organs (bones, lungs, liver, brain), almost half of all metastases manifest themselves first in the bone and then appear in other organs, raising the possibility of secondary metastatic dissemination from a primary bone site [265–267]. In animal model systems, it was shown that the bone tissue microenvironment promotes the ability of metastatic cells (of BC or prostate cancer origin) to further spread horizontally, giving rise to secondary metastases [268]. This is associated with epigenetic reprogramming that imparts EMT features and endocrine resistance to the BC metastatic cells departing from the bone lesions, by a mechanism shown to be distinct from clonal, mutation-driven selection [268, 269].

In more metabolically oriented approaches, it was shown that BC cells metastasizing to different organs exhibit different metabolic programs [270]. In particular, liver metastases displayed increased aerobic glycolysis and reduced OXPHOS or glutamine metabolism *vs*. lung or bone metastases. This was mechanistically linked to increased HIF-1α activity and consequent increase of expression of one of its critical target genes, pyruvate dehydrogenase kinase-1 (PDK1), that antagonizes the function of pyruvate dehydrogenase (PDH), a key rate-limiting enzyme for pyruvate conversion to acetyl-CoA, and entry into the TCA cycle. Importantly, PDK1 levels were elevated in liver metastases in BC patients *vs.* their primary tumors [270]. Conversely, BC lung metastases displayed increased pyruvate carboxylase (PC)-dependent anaplerosis, leading to conversion of pyruvate into oxaloacetate, when compared to their primary tumors [271]. This might to be due to increased availability of pyruvate in the lung microenvironment as, in healthy mice, the ratio of pyruvate/glutamine was three times higher in the interstitial fluid of the lungs compared to the levels in the blood plasma [271]. Finally, brain metastases of BC displayed elevated de novo FA synthesis (FASN dependent) as a result of adaptation to decreased lipid availability in the brain relative to other tissues [134].

The sum of the above argues for different metabolic requirements to sustain metastatic growth of BC cells in various organs, possibly as a function of metabolic plasticity/adaptation to varying nutrient availability at the various metastatic sites. In addition, the results in the bone setting suggests an active role of the tissue microenvironment in supporting horizontal metastatic spreading: a phenotype likely associated with awakening from dormancy, albeit not metabolically defined yet.

Despite this progress, the picture remains unclear. One major problem resides in the nature of the available metastatic models. These rely mostly on the transplantation in mice of BC cell lines or of patient-derived xenograft (PDXs) cells, which are implanted subcutaneously or orthotopically in the mammary gland and then monitored for tumor growth and appearance of metastasis. Firstly, the starting material of these experiments is already a less-than-adequate representation of the events under study. Even when PDXs are used, the cells are derived from tumors that were removed because of their clinical detectability, which is—in all probability—way past the moment in which BCs metastasize [185–187]. Secondly, immunocompromised recipient mice must be used for studies with human material, abrogating one of the essential components of the cancer–TME interaction. Conversely, when isogenic systems are used, the loss of the natural genetic variability of human samples might minimize or attenuate the non-mutational component of the interactions. Thirdly, and perhaps more worryingly, the entire time kinetics of the events is altered in these models. Xenografted mice typically develop vigorous metastases within weeks/months not years from the “appearance” of the primary tumor. Are these metastases a faithful representation of the metastases that we see in real BC patients? A discussion of this issue is beyond the scope of this review (see however [272] for an account of available laboratory models of metastasis in BC). It seems clear, however, that while available model systems have provided enormous value, they might be reaching their limit of resolution.

A recent study described an approach to study metastatic BC to the brain in a closer-to-reality representation [273]. The study focused on HER2 + BC, which are known to preferentially metastasize to the brain (together with TNBC) with respect to Luminal BCs. After transplantation of HER2 + BC cell lines, brain metastases were excised from the brain and re-established in culture for characterization. Three types of metastatic cells were characterized: synchronous (S-met, from mice displaying overt lesions within 5 weeks), latent (L-met, from mice with no evident clinical lesions after 3 months), and metachronous (M-met, from mice displaying overt lesions after 2–3 months). Phenotypic and metabolic characterization of these cells revealed differences in metabolic plasticity which was associated with metastatic fitness. Data were compatible with a model in which: (i) DTCs that could not compete with brain-resident cells for glucose utilization and could not metabolize glutamine would perish; (ii) cells that could utilize glucose efficiently and metabolize lactate would initiate S-met; (iii) cells that could utilize glutamine as an alternative to glucose would survive as L-met; and (iv) L-met would then adapt to the metabolic environment giving rise to M-met [273].

## Adipocyte–cancer interactions in BC

There is a compelling link between obesity, dysregulation of cellular metabolism, and BC. This is part of a vast involvement of obesity in cancer; the International Agency for Cancer Research has established strong evidence that connects obesity with 13 different types of cancer, including post-menopausal BC [274]. Indeed, obese women (BMI, body mass index ≥ 40) are twice as likely to die from BC as women with a normal BMI [275]. In BC, the impact of adipocytes on tumorigenesis depends on two intertwined aspects: (i) the systemic effects of obesity, due to the secretory/endocrine nature of the adipose tissue [276], and (ii) local effects in the BC microenvironment, due to the close proximity of the epithelial and stromal (mostly adipocytes) components in the mammary gland, with a remarkable numeric predominance of the latter. Indeed, during the development of BC, invasion of the mammary stroma results in contacts between cancer cells and adipocytes, and even marginal invasion of the adipose tissue correlates with worse prognosis [277, 278].

**Fig. 7:**
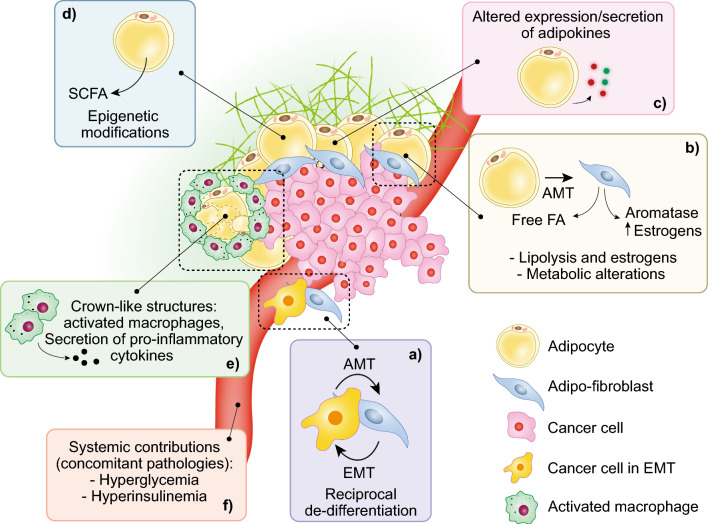
Adipocytes and breast cancer. The major reciprocal interactions between breast adipocytes and BC cancer cells are illustrated. a A feed-forward loop between cancer cells and resident adipocytes causes de-differentiation. Cancer cells undergo EMT, while adipocytes undergo AMT, becoming adipo-fibroblasts. b Adipo-fibroblasts display increased lipolysis and release FA that can be utilized by cancer cells; they also trigger a “reverse Warburg” effect secreting high-energy metabolites that are utilized by cancer cells. Adipo-fibroblasts also express high levels of aromatase, thus altering estrogen metabolism. c Adipocytes and adipo-fibroblasts display altered patterns of secretion of adipokines and various other signaling molecules. d Adipocytes and adipo-fibroblasts produce SCFA that alter the epigenetic regulation of cancer cells and other stromal cells in the TME. e Macrophages are also activated in the TMA. The morphological counterpart of this activation is represented by “crown-like structures,” constituted by dying (or dead) adipocytes surrounded by macrophages. In these structures, macrophages are switched toward a M1 pro-inflammatory state. **f** Finally, obesity-related hyperglycemia and hyperinsulinemia further provide stimuli for cancer cell growth

Even though adipocytes are abundant in the breast (9:1 ratio to epithelial cells), the crosstalk between adipocytes and BC cells has received comparatively less attention *vs*. the interactions between cancer cells and CAFs or macrophages. However, important advancements have been recently made. In particular, the effects of hormones, chemokines/growth factors, and adipokines secreted by adipocytes (including estrogens, leptin, adiponectin, resistin, oncostatin-M, lipocalin-2, IL6, IL1b, TNFα, HGF, ECM-degrading proteases) on the cancer epithelial component have been studied (reviewed in [10, 11] and summarized in Fig. 7). The complex network of interactions established in the cancer ecosystem results in cancer-favorable phenotypes: (i) induction of a pro-tumorigenic inflammatory state, (ii) stimulation of proliferation and of a migratory/invasive phenotype, (iii) reciprocal de-differentiation accompanied by acquisition of mesenchymal traits, and (iv) metabolic reprogramming [10, 11]. While most studies have focused on white adipose tissue, an emerging role for brown adipose tissue (BAT) and other types of adipose tissues is also coming into focus [10]. In the case of BAT, for instance, it was recently shown, in mouse models, that BAT activation by cold exposure could effectively decrease the growth of several tumors, including BC, by competing for glucose availability, with ensuing reduced glucose utilization and aerobic glycolysis in the tumor [279].

For the purposes of this review, we will focus on the emerging notion of the adipocyte–BC cell interactions as inducers of reciprocal de-differentiation and acquisition of mesenchymal characteristics, which sits at the heart of a complex metabolic subversion in the BC TME.

ER + BCs represent the majority of BCs and they predominantly occur after menopause [280]. This is apparently a bit of a paradox, considering that estrogen levels decrease upon menopause. The paradox is resolved if one considers the local production of estrogens. Indeed, while post-menopausal women have circulating levels of estrogens 10–15 times lower than pre-menopausal ones, the hormonal levels in the breast gland are similar [281]. In addition, obese post-menopausal women have higher levels of circulating estrogens than non-obese ones [282]. The combination of systemic (due to obesity) and local (due to the peculiar structure of the mammary gland, and worsened by obesity) effects on estrogen production drive the proliferation of estrogen-dependent malignancies, uterus and breast, and are directly due to the activity of the adipose tissue, which is the major producer of estrogens after menopause. This is due to the expression of aromatase by adipocytes. Aromatase (CYP19A1) is an enzyme that converts 19-carbon steroids (androgens, such as androstenedione and testosterone) to 18-carbon steroids (estrogens, such as estrone and estradiol) [283]. Aromatase is induced in adipocytes by pro-inflammatory factors (such as IL6, TNFα) and adipokines (such as leptin), secreted by the adipocytes themselves, or by other cytotypes in the TME, including inflammatory cells and cancer cells themselves [284]. In obesity, as the fat mass increases, the aromatase levels and the resulting estrogen levels rise. Thus, it is not surprising that the strongest link is between obesity and ER + BC vs. other BC subtypes [285].

This latter evidence, however, confronts us with a substantial paradox: why is the ER + BC risk increasing with obesity after menopause, but not before, considering that there is abundance of circulating estrogens before menopause? In actual fact, the situation is even more complex, since pre-menopausal obese women may display a modest reduction in the risk of BC vis-à-vis a sizable risk increase after menopause [286]. The answer might lie in the type of produced estrogen, as evidenced by recent studies of the Slingerland’s group. In pre-menopausal women, the major estrogen is the ovarian-produced 17β-estradiol (E2); post-menopause, estrone (E1)—produced by adipocytes from adrenal androstenedione—becomes the prominent one [287]. It was shown that E1 differs fundamentally from E2 in at least two respects: (i) it induces inflammation through NFκB, while E2 opposes it; (ii) it induces EMT in the epithelial cancer component and metastatic ability, through transcriptional activation of the SNAI2 transcription factor, while E2 represses it [288, 289].

The acquisition of mesenchymal-like phenotypes is involved in BC progression not only through the EMT of epithelial cells but also through mesenchymal transition of adipocytes (AMT). Aromatase is expressed in immature adipocytes, frequently referred to as adipo-fibroblasts, but not by mature ones [284, 290]. It has been shown that the interaction between epithelial BC cells and resident breast adipocytes can drive the mesenchymal transition, de-differentiation, of the latter. In turn, the de-differentiated adipo-fibroblasts contribute to the TME through inflammation and ECM remodeling. Furthermore, they display distinct metabolic features *vs*. mature adipocytes, thereby contributing to the creation of a tumor-friendly microenvironment [291]. Although not directly tested in the mentioned study, it is likely that de-differentiated adipocytes also contribute to increased E1 levels, with the consequences reviewed above.

In another study, Onuchic et al. have shown that AMT is frequent in the most aggressive forms of BCs [292]. This is in agreement with previous data showing different capacities of distinct tumor types to induce AMT [293, 294]. Furthermore, they showed that a less adipose stroma displays lower levels of mitochondrial activity and is associated with cancer epithelial cells with higher levels of OXPHOS [292]. This is consistent with the concept of metabolic coupling or the reverse Warburg effect [22], in which cancer cells stimulate anaerobic glycolysis in the surrounding stroma, possibly by inducing AMT (adipo-fibroblasts are more glycolytic than adipocytes [295]). In turn, glycolytic adipo-fibroblasts would secrete high-energy metabolites (e.g., pyruvate, lactate), thereby sustaining the TCA cycle and OXPHOS metabolism in epithelial cancer cells [22].

The metabolic connection between de-differentiated adipocytes and cancer cells extends beyond the regulation of glycolysis/OXPHOS. Adipocytes undergoing AMT activate lipolysis and lose their lipid droplets [293]. In ovarian cancers, it was shown that the FA produced as a result of lipolysis is captured by cancer cells where they are used for energy production through FAO [296]. The same is true in the case of co-cultures of adipocytes and BC cells [297]. In this latter case, quite surprisingly, FAO is apparently uncoupled from ATP production and instead induces activation of the AMPK/acetyl-CoA carboxylase circuitry, which does not lead to increased proliferation/survival but rather supports migratory/invasive behavior [297].

An additional level of complexity was unveiled by recent studies on creatine metabolism. Creatine is phosphorylated in the cell by creatine kinase(s) (CK) yielding phospho-creatine, which in turn serves as a high-energy phosphate reservoir, as it works as a phosphate donor in the conversion of ADP into ATP, catalyzed also by CK [298]. The relevance of phospho-creatine to meeting the energy demands of BC cells is underscored by the observation that, in HER2 + BCs, CK is stabilized/activated, through HER2-mediated tyrosine phosphorylation [299]. In a recent study, on an obesity accelerated model of BC, a transcriptomic analysis revealed marked overexpression of glycine amidinotransferase (GATM) in cancer adipocytes, but not in normal (contralateral) ones [300]. GATM is the rate-limiting enzyme in creatine biosynthesis in adipocytes, and adipose-selective ablation of GATM attenuated tumor growth. The major transporter of creatine into epithelial cells is SLC6A8, the silencing of which also reduces tumor growth [300]. Finally, GATM or SLC6A8 overexpression in human BCs predicted aggressive disease course [300]. These results strongly link creatine secretion by cancer adipocytes to sustained energy metabolism in cancer epithelial cells, although the mechanism responsible for GATM overexpression in adipocytes has not been investigated.

Mammary adipocytes can also contribute to epigenetic reprogramming of the epithelial component of BCs. It was shown that mammary cancer adipocytes secrete β-hydroxybutyrate, which is alone capable of supporting the growth of mammary tumors in in vivo xenograft models [301]. This effect is likely mediated by the β-hydroxybutyrate-exerted inhibition of histone deacetylases (HDACs) [302], which results in increased histone H3K9 acetylation and upregulation of several tumor-promoting genes [301]. While it is not known whether the production of β-hydroxybutyrate by cancer adipocytes is linked to AMT, these results can also be viewed in the framework of the emerging relevance in BC of the metabolism of short-chain fatty acids (SCFAs, the family to which β-hydroxybutyrate belongs). This topic is further discussed in the next section.

In conclusion, the sum of the above argues for the existence of a BC microenvironment in which reciprocally induced mesenchymal transitions, in adipocytes and cancer cells, create a cancer-friendly metabolic TME to which adipocytes participate with various inflammation-driving adipokines, estrogens, ECM modifications, and metabolic reprogramming. It remains to be established how the various levels of metabolic reprogramming induced by the adipocyte-cancer cell interactions are integrated in space and time, in the various phases of tumor growth and its natural history. It is relevant to note, however, that the de-differentiation of adipocytes into adipo-fibroblasts is likely to involve an intrinsic plasticity of mature adipocytes. Indeed, it was shown that mammary adipocytes can de-differentiate into adipo-fibroblasts during pregnancy/lactation, to make place for alveolar-epithelial cells, and can also re-differentiate upon weaning [303]. Thus, similarly to what we have discussed for EMT, it seems that a metabolic cancer-friendly environment is built through the co-optation of defined, physiologically relevant, programs, rather than through cancer-specific rewiring or reprogramming of metabolic functions.

## Microbiota–cancer interactions in BC

A multitude of microorganisms, collectively termed the microbiota—and including bacteria, viruses and fungi—inhabits bodily surfaces contributing to the creation of symbiotic ecosystems. The host–microbiota symbiosis is involved in the execution of physiological functions, and it has been studied predominantly in the gut, where it participates in bodily metabolic homeostasis [304, 305]. Accordingly, alterations in the microbiota (dysbioses) can lead to a host of diseases [306]. The pioneering findings on the causal role of *Helicobacter pylori* in gastric cancer [307] paved the way for a wealth of studies showing that alterations in the microbiota are relevant to cancer [308, 309]. Evidence emerged initially from studies on colon cancer [310, 311] and were then extended to other types of cancers, including BC [312, 313]. Today, alterations of the microbiota are considered an emerging hallmark of cancer [2]. While the impact of dysbioses on cancer has been studied mostly in connection with bacteria, a role for fungal microbiota is also emerging [314–316]. In addition, an ever increasing—and largely unexplored—gut virome is involved in diseases and possibly in cancer [317].

**Fig. 8:**
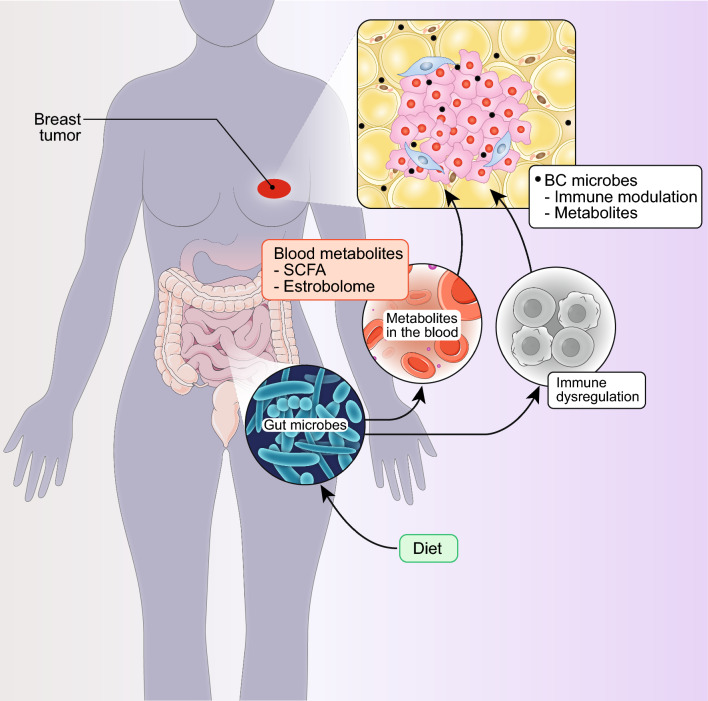
The microbiota in breast cancer. The major influences of the gut microbiota and of the local breast microbiota (both extracellular and intracellular) on the tumor ecosystem are show, as described in the main text

Subverted microbiota can contribute to carcinogenesis through multiple mechanisms (see [2] and Fig. 8), among which dysregulation of local and systemic immunity, frequently associated with a pro-inflammatory state, and alterations of bacterial metabolites or release of bacterial products [318–321]. A spectacular demonstration of this latter concept was provided by Kadosh et al. who showed that in mouse models of intestinal cancers, the gut microbiota can switch some mutant forms of p53 from tumor suppressive to oncogenic. Surprisingly, the entire effect of the gut microbiome could be recapitulated by a single, gut microbiota-derived, metabolite, gallic acid [322].

Dysregulated immunity and bacterial metabolism are also intimately connected. For instance, the gut microbiota uses bile acid metabolites to send messages to the liver to control the accumulation of natural killer cells, via a chemokine-mediated mechanism, hence controlling anti-tumoral activity [323]. Finally, an important role for bacterially produced SCFAs, including acetate, propionate and butyrate, is known. These metabolites are produced by a host of intestinal bacteria through the metabolism of indigestible dietary fibers [324] and act as local or long-distance signaling molecules mostly through their interaction with G-protein-coupled receptors [325]. They perform a paramount function in gut homeostasis by (i) restricting the colonization by other, potentially pathogenetic, microbes, (ii) maintaining the gut epithelial barrier and its role in local and systemic immunity, and (iii) protecting against bowel inflammation [324]. Unsurprisingly, therefore, the reduction of the SCFA-producing microbiota is associated with colon cancer [326] as well as other cancers (see below). However, the picture is not completely clear, as some studies have reported that SCFAs (in particular, butyrate) drive tumorigenesis [327, 328]. This is known as the butyrate paradox which might be resolved invoking different effects of this molecule as a function of its concentration [329], although also this explanation has been challenged [330, 331]. Of note, one of the recognized functions of butyrate is to inhibit HDACs, thus modifying the cellular epigenetics [302, 332]. The paradoxical effects of butyrate, therefore, might simply reflect its impact on different cell-specific epigenetics landscapes.

From the cancer metabolism viewpoint, the sum of this evidence represents quite a paradigm shift, as it compels us to view metabolic plasticity not just in terms of the cancer ecosystem or of the organismal ecosystem, but in terms of super-organismal ecosystem, microbiota, and host, in which a constantly changing microbiota landscape, and the linked microbial metabolism, might have profound impacts on several steps of the natural history of cancer and on therapeutic responses.

In the remainder of this section, we will focus on some of the features of the interaction between the microbiota and the mammary gland of relevance to BC.

In BC, we can schematically distinguish three levels of alterations of the microbiota as relevant to tumorigenesis:

(i) The role of the gut microbiota
(ii) The role of the mammary gland microbiota.
(iii) The role of the intracellular tumor microbiota

*The role of gut microbiota in BC* There is evidence that the gut microbiota of BC patients is different from that of healthy individuals [333–335]. Metagenomic comparisons of the relative microbiomes (with microbiome, we mean the collection of genes of a given microbiota) revealed loss of bacteria producing certain metabolites, in particular SCFAs, in BCs [333]. In xenograft mouse models of BC, it was shown that antibiotic-induced modifications of the gut microbiota caused increased stromal fibrosis and infiltration of macrophages or mast cells, associated with augmented tumor growth and metastatic ability [336, 337]. Deep sequencing and metagenomic analysis of fecal material in these animals revealed loss, upon antibiotic treatment, of several commensal species. Restoration of one of these, *Faecalibaculum rodentium*, normalized tumor growth to control levels [337]. Interestingly, transcriptomic analysis of tumors revealed predominantly alterations in metabolic processes, including protein and lipid metabolism. This was paralleled by a significant loss of microbial metabolites (again SCFAs) in cecal content [337]. In clinical studies, antibiotic treatment was associated with reduced efficacy of neoadjuvant chemotherapy and poorer prognosis in BC [338], and reduced response to immune checkpoint inhibitors in several epithelial malignancies, including BC [339].

Finally, gut bacteria might directly influence the growth of BC by interfering with estrogen catabolism through the action of several gene products, globally referred to as the estrobolome [340], among which bacterial β-glucuronidase is paramount. Estrogens, metabolized in the liver in conjugation with glucuronic acid are then excreted in the bile. Excessive presence of β-glucuronidase-producing bacteria in the gut de-conjugates estrogens, which can be re-absorbed and enter the circulation, targeting hormone-sensitive organs, such as the breast [312, 341]. The extent to which a subverted estrobolome might participate in BC initiation or progression, or even to acquired resistance to endocrine therapy [342], is not known and will certainly represent an active area of investigation in the future.

The gut microbiota is not solely composed of bacteria. In a recent study, the role of the fungal gut microbiota in the tumor response to radiotherapy was explored in mouse models of BC [343]. Bacteria and fungi had an opposite impact: gut bacteria were needed to sustain the therapeutic response, while gut fungi had a detrimental effect, possibly by opposing the mounting of an efficacious anti-tumor immune response following radiotherapy. Depletion of bacteria, through antibiotics, led to expansion of the fungal population, adding to the evidence that depletion or alteration of the bacterial gut microbiota might negatively impact on the natural history of BC at several levels, including therapy response. Intestinal fungi can impact cancers “at a distance” through their ability to regulate not only intestinal mucosal immunity but also systemic immunity [344]. Another possibility could be envisioned, as fungi are known to shed components in the bloodstream. One such component, β-glucan, interacts with CLEC7A (a.k.a., Dectin-1), a lectin overexpressed by BCs [343] and might therefore modulate BC cells at a distance.

*The role of the mammary gland microbiota* In addition to the gut, microbes are present on virtually every surface of the body and also in tumors [345, 346], where they contribute to tumorigenesis through different mechanisms [347, 348]. Despite having long been considered sterile, the mammary gland possesses a rich microbiota [349], subject to variations as a function of multiple variables, including diet. In a primate animal model, different diets (Mediterranean *vs*. Western) led to important variations of breast microbiota and content of microbial breast metabolites [350]. This might be relevant to cancer since (i) Mediterranean diet was associated with increased presence of *Lactobacillus* [350], (ii) in humans, BCs display decreased *Lactobacillus *vs. non-malignant breast tissues [351], and (iii) Mediterranean diet protects from BC [352, 353]. Thus, diet might contribute to BC also through induced modifications of the resident microbiota. Indeed, metagenomic analysis revealed that cancers possess different organ-resident microbiota and that BCs display the richest and most diverse microbiome among all cancers [345, 346]. Furthermore, there is evidence for different microbial populations being prominent in different BC subtypes and associated with prognostic outcome [92, 354].

*Fusobacterium nucleatum* is a common oral resident, known to be associated with periodontal diseases, which has been implicated in colorectal cancer [355]. *F. nucleatum* is thought to enter the bloodstream during bacteremic episodes, which are frequent in periodontal diseases, and to lock to colorectal tissues through a surface lectin, Fap2 [356]. A similar situation might occur in BCs, since *F. nucleatum* is present in malignant breast tissues but not in normal ones [351]. Indeed, in mouse models of BC, it was shown that *F. nucleatum* increases tumor growth and progression, probably through suppression of the T cell-mediated immune response [357]. While the mechanism of action of *F. nucleatum* in breast carcinogenesis is not completely clear, the mechanisms of its targeting to BC cells involve a clear metabolic component, as it was shown that BC cells display higher surface levels of the sugar Gal-GalNAc [357]. Thus, alterations in sugar metabolism in the tumor modify the ecosystem, allowing cancer–bacteria interactions.

A direct connection between bacterial metabolism and cancer behavior (in this case, response to immunotherapy) was provided by studies on TNBC. These tumors can be classified, based on genomic and transcriptomic analyses, as immune-modulatory (IM, associated with an immune activated TME) and non-IM: the IM subtype displays better response to immune-therapy [358]. IM-TNBC displayed higher abundance of *Clostridiales* and its metabolite trimethylamine N-oxide (TMAO). Accordingly, patients with high levels of plasma TMAO responded better to immune-therapy as TMAO activates CD8 + T cell-mediated anti-tumor immunity [359]. Mechanistically, TMAO could be directly linked to the anti-tumor immune response, since the metabolite induced pyroptosis (a form of inflammatory cell death capable of evoking an anti-tumor immune response) of BC cells through activation of the endoplasmic reticulum stress kinase, EIF2AK3 (eukaryotic translation initiation factor 2 alpha kinase 3, a.k.a. PERK) [359]. Thus, bacterial metabolites can contribute to anti-cancer responses not only through the modulation of inflammatory/immune cells but also through direct effects on epithelial cancer cells.

Fungal microbiota might also play a role in BC. A recent Pan-cancer analysis revealed that distinct fungal–bacterial ecosystems characterize different cancers. In BC, the predominance of the fungus *Malassezia globosa* was associated with worse clinical outcome [315]. While the mechanism is not known, it is notable that the same fungus is involved in pancreatic cancer. In this latter type of cancer, fungal sugars bind to surface lectins on pancreatic cells (among which CLEC7A, mentioned above) activating signaling pathways leading to IL-33 secretion or to activation of the complement cascade modulating local inflammatory and immune responses [360, 361]. These findings further underscore the impact of altered lectin-mediated recognition in the triggering of cytokine-dependent signaling pathways in BC.

*The role of the intracellular tumor microbiota in BC* Bacteria not only thrive on mucosal surfaces but can also invade cancer cells and become permanent intracellular residents [362, 363]. A striking confirmation of the importance of intracellular bacteria derived from a recent paper by Fu et al. [364]. The authors demonstrated that, in a mouse model of BC, intracellular bacteria can drive metastasis apparently without affecting the growth of the primary cancer. Mechanistically, this was linked to increased resistance to shear stress due to bacterially induced actin cytoskeleton remodeling. Several remarkable questions arise from these findings. First, the growth of the primary tumor was not affected by the cancer microbiota, only its metastatic ability. So, what is the advantage-conferring property of tumor intracellular bacteria? Second, the growth of the primary tumor was affected by the composition of the gut microbiota, similarly to other reviewed findings. This indicates that two microbiota, from gut and breast, participate in distinct phases of the natural history of BCs: something that might have therapeutic implications. Third, the microbiomes of BCs were rather different from that of adjacent normal breast tissue [364]. So, where are the different bacteria coming from? Finally, while the microbiomes of BC-derived early lung metastatic lesions were similar to those of the primary tumor, those of frank lung metastases were more heterogeneous, indicating some level of selection. Answers to these questions will substantially advance our knowledge of how the interactions between different microbiota and the host cancer components (epithelial and stromal) shape the natural history of BCs.

The distinctive features of the breast microbiota could be exploited to predict prognosis and identify unique targetable vulnerabilities. Because the microbiota is known to significantly affect response to treatment also supporting drug resistance, the possibility to manipulate its composition to improve therapeutic efficacy and reduce toxicity represents an attractive perspective that is currently being extensively investigated [365].

## Outlook: metabolism as a target in BC

The prospect of targeting metabolic alterations in cancer is receiving considerable attention, at the experimental and clinical levels. A number of drugs, widely used in clinical oncology, are indeed metabolic drugs, such as 5-Fluorouracil, Hydroxyurea, Gemcitabine, and Methotrexate, which target—with various mechanisms—nucleotide metabolism. In the modern era of targeted therapies, an important proof of principle that other relevant metabolic pathways can be successfully targeted as well came from studies of IDH1 and IDH2 [63–65, 366]. Two inhibitor drugs, Enasidenib and Ivosidenib, targeting IDH1 and IDH2, respectively, were FDA approved for treatment of AML; the indication for Ivosidenib was extended to cholangiocarcinoma in 2023. Interestingly, the mutations in IDH1 and 2 do not impact cancer metabolism per se, as they rather cause the appearance of a neomorphic activity of IDH that perturbs the physiological oxidative decarboxylation of isocitrate to α-KG (see Fig. 4) and promotes the reduction of α-KG to D-2-hydroxyglutarate (D-2HG). In turn, D-2HG inhibits histone demethylases and the TET family of 5-methylcytosine hydroxylases, resulting in extensive epigenetic reprogramming [367, 368].

As of today, essentially every known metabolic pathway involved in cancer is being targeted in preclinical studies, including aerobic glycolysis, glutamine metabolism, FA metabolism, nucleotide metabolism, and various mitochondrial functions [369]; a few of these compounds have reached the level of clinical studies [370]. In addition, the evidence that oncometabolites, such as D-2HG or lactate, impact on cellular epigenetics is stimulating research into epigenetics drugs as modifiers of cancer metabolism [371]. Finally, considerable efforts are being directed at cancer repurposing of metabolic drugs approved for other pathologies, with the intent of lowering costs, and reducing risk failure in early phases of clinical experimentation (see for instance [372], also reviewed in [373]).

From a biological viewpoint, the idea of metabolic targeting cancers offers intertwined advantages and drawbacks. On one hand, it is reasonable to think that the number of metabolically altered pathways might be sizably less numerous than the mutational repertoire of cancers. In other words, targeting a metabolic phenotype might be efficacious in several cancers harboring alterations impinging on that pathway. At the same time, therapy resistance to targeted drugs might be circumvented by metabolic drugs, if the metabolic phenotype is intercepted at a distal enough point in the signaling pathway in which the mutational escape occurs. Obviously enough, the enormous metabolic plasticity of cancer might represent a serious obstacle, if the cancer cell simply switches its addiction to a different metabolic pathway. In this perspective, the use of metabolic mono-therapy, or its simple association to traditional chemotherapy or targeted therapy, might not be sufficient and simultaneous targeting of several metabolic pathways might be required. In addition, the evidence that cancer cells and their TME do not reprogram their metabolism, but rather coopt metabolic programs present also in physiological conditions, predicts important toxicity of anti-metabolic drugs. This might be one of the reasons why so few metabolic drugs have been approved. Indeed, one should keep in mind that anti-IDH1 and 2 drugs target a neomorphic, i.e., cancer-specific, activity.

With these difficulties in mind, one approach appears particularly promising, as there is ample evidence that alterations in the TME lead to metabolic immune suppression of effector cells and promote regulatory immune cells [115]. Thus, the use of anti-metabolic drugs to remove the cancer TME-induced blockade of immune response, thereby unleashing the power of immunosurveillance, might prove advantageous, also in light of the possibility of restoring immune checkpoint blockade in tumors unresponsive to immunotherapy. A striking proof of principle for this possibility was recently provided by the demonstration that, in animal models, a glutamine antagonist suppresses oxidative and glycolytic metabolism of cancer cells, while enhancing oxidative metabolism and inducing an activated phenotype in effector T cells [374]. It is of note that in this study, rather than targeting glutaminase, the authors employed a derivative of a molecule 6-diazo-5-oxo-l-norleucine known to inhibit a broad range of glutamine-requiring enzymes [375], supporting the general idea that interfering in a “global” fashion with a metabolic phenotype might represent a winning therapeutic strategy.

With specific regard to BC, the most pressing need is for the therapy of metastatic disease, even before it becomes clinically evident. Much research on metastasis is focused on how cells metastasize, and most experimental read-outs assess “prevention” of metastases. The parallel model of metastasis and the phenomenon of dormancy argue that refocusing our efforts on the mechanism of metastatic dormancy might teach us important lessons on how to keep metastases dormant. An area in which metabolic drugs might play an important role.

## Data Availability

Not applicable, this manuscript is a review.
